# Identifying Personality Characteristics and Indicators of Psychological Well-Being Associated With Attrition in the Motivation Makes the Move! Physical Activity Intervention: Randomized Technology-Supported Trial

**DOI:** 10.2196/30285

**Published:** 2022-11-25

**Authors:** Kaisa Kaseva, Mari Tervaniemi, Enni Heikura, Kaisamari Kostilainen, Maritta Pöyhönen-Alho, J Kevin Shoemaker, Robert J Petrella, Juha E Peltonen

**Affiliations:** 1 Department of Sport and Exercise Medicine, Clinicum Faculty of Medicine University of Helsinki Helsinki Finland; 2 Cicero Learning Faculty of Educational Sciences University of Helsinki Helsinki Finland; 3 Cognitive Brain Research Unit Department of Psychology and Logopedics University of Helsinki Helsinki Finland; 4 Obstetrics and Gynecology University of Helsinki and Helsinki University Hospital Helsinki Finland; 5 Neurovascular Research Laboratory School of Kinesiology Western University London, ON Canada; 6 Division of Sport and Exercise Medicine Department of Family Practice University of British Columbia Vancouver, BC Canada; 7 Center for Studies in Family Medicine Schulich School of Medicine and Dentistry Western University London, ON Canada; 8 Clinic for Sports and Exercise Medicine Foundation for Sports and Exercise Medicine Helsinki Finland

**Keywords:** randomized trial, physical activity, lifestyles, personality, psychological well-being, study attrition, mental health, lifestyle interventions

## Abstract

**Background:**

Data attrition has been a common problem in longitudinal lifestyle interventions. The contributors to attrition in technology-supported physical activity interventions have not been thoroughly studied.

**Objective:**

The present study examined the roles of personality characteristics and indicators of psychological well-being in data attrition within a technology-supported, longitudinal intervention study with overweight adults.

**Methods:**

Participants (N=89) were adults from the Motivation Makes the Move! intervention study. Data attrition was studied after a 3-month follow-up. Participants’ personality characteristics were studied using the Short Five self-report questionnaire. Psychological well-being indicators were assessed with the RAND 36-item health survey, Positive and Negative Affect Schedule, and Beck Depression Inventory. Logistic regression analyses were conducted to assess the risk of discontinuing the study. The analyses were adjusted for sex, age, study group, and educational status.

**Results:**

At the 3-month follow-up, 65 of 89 participants (73% of the initial sample) had continued in the study. Participants’ personality characteristics and indicators of psychological well-being were not associated with the risk of dropping out of the study (all *P* values >.05). The results remained the same after covariate controls.

**Conclusions:**

Participant attrition was not attributable to personality characteristics or psychological well-being in the Motivation Makes the Move! study conducted with overweight adults. As attrition remains a challenge within longitudinal, technology-supported lifestyle interventions, attention should be paid to the potentially dynamic natures of personality and psychological well-being, as well as other elements beyond these.

**Trial Registration:**

ClinicalTrials.gov NCT02686502; https://clinicaltrials.gov/ct2/show/NCT02686502

## Introduction

Cardiovascular diseases are the leading cause of premature death worldwide. Overweight and obesity have been recognized as among the most severe risk factors for these diseases. According to the World Health Organization, more than 1 billion people worldwide are overweight (BMI ≥ 25), and more than 300 million are obese (BMI ≥ 30) [1]. Overweight and obesity may also be associated with comorbidities, referring to a person’s vulnerability to other illnesses and diseases. Comorbidity may also refer to interactions between illnesses that can worsen the course of both. For instance, due to the possibility of unprecedented outbreaks of infectious diseases (eg, COVID-19), it is of utmost importance to pay attention to the prevention of risk factors, such as obesity, that are likely to contribute to the development of comorbidities.

Along with genes, unhealthy lifestyles are among the most prominent contributors to overweight and subsequent negative health outcomes. Physical inactivity has been recognized as one of the primary contributors to overweight and obesity [2,3]. Recently, it has been suggested that even small improvements in physical fitness reduce the risk of coronary heart disease and that any amount of physical activity is beneficial for overall health [4]. Despite this knowledge, the prevalence of obesity has increased during the last 3 decades, and therefore, there is a need to pay attention to, as well as target research resources toward, physical activity in preventing and hindering the epidemic of obesity [5].

Recently, many physical activity and exercise promotion actions and interventions have been designed to prevent the development of obesity and other health risk factors. Some physical activity interventions aiming to increase healthy physical activity habits among overweight people have demonstrated favorable results, but equivocal results regarding expected outcomes also exist [6]. Recently, technological innovations have been regarded as promising tools to optimize the effectiveness of interventions by improving intervention delivery and adherence to participation [7,8]. In addition, eHealth and mobile health (mHealth) technologies allow collection of reliable, comprehensive, and diverse information about the users’ physical activity and can reach large populations quickly [9-11]. Many technology-based health interventions have proved to be efficient in increasing physical activity, contributing to weight loss, and improving overall health [12,13].

Along with the creation of valid study designs, the effectiveness of research is related to participants’ commitment to the completion of studies. Attrition in longitudinal studies and interventions refers to participants dropping out of the study before its completion or stopping use of the program or application during participation [14]. High attrition is a common issue in longitudinal physical activity interventions and a major challenge to the validity of the research [15,16]. In addition to a variety of individual factors, psychological qualities have been suggested to be important contributors to health behaviors, as well as to participation in and commitment to interventions [17,18]. Further assessment of these factors could improve the effectiveness of interventions [17].

Personality characteristics have been regarded as among the most essential contributors to behavioral choices [19]. Personality reflects individual differences in thinking, feeling, and behaving, and it matures through age. In the widely acknowledged 5-factor model of personality (ie, the “Big Five” model), personality characteristics are categorized into 5 broader continuums [20,21]. Individuals scoring high in neuroticism tend to experience negative emotions, such as anger, fear, and stress. In contrast, individuals high in extraversion tend to exhibit positive emotions, including cheerfulness and enthusiasm. Conscientious individuals have been characterized as diligent, organized, and disciplined, and display planned, rather than spontaneous, behaviors. Individuals high in openness to experience are open-minded and creative, whereas those high in agreeableness tend to exhibit kindness, cooperativeness, and positivity in interpersonal relations [20]. The psychobiological theory of personality has introduced a 7-factor model, including 4 dimensions of temperament (novelty seeking, harm avoidance, reward dependence, and persistence) that are regarded as individuals’ inherited, reactive tendencies, and 3 dimensions of character (self-directedness, cooperativeness, and self-transcendence) that mature through age [22,23]. Both the 5- and 7-factor paradigms of personality have also been applied within recent physical activity interventions.

High levels of openness to experience, as well as hedonistic orientation, have been shown to relate favorably to attitudes toward health behavior interventions [24,25]. Reward dependence, which correlates highly with extraversion and agreeableness [26], has been shown to play a role in determining high success rates in therapy-induced weight management [27]. Persons who were successful at achieving weight loss in a weight-loss program had a lower novelty-seeking trait, which correlates positively with conscientiousness [23], than those who did not reach their goals [28]. Personality seems to also play a role in becoming interested in participating in technology-based interventions [29], but more evidence on personality’s role in the commitment to such interventions is needed.

Along with personality, the level of psychological well-being, referring to a person’s experience of their own well-being, is associated with the commitment to a healthy lifestyle, as well as the commitment to lifestyle interventions [18,30]. The concept of health-related quality of life refers to a person's evaluation of their physical and mental health over time [31], and it can be regarded as one of the most essential contributors to psychological well-being. Past research has demonstrated that higher levels of health-related quality of life also contribute to increased physical activity [32]. Comparably, studies have indicated that experiencing limitations related to physical and psychological functionality, negative thinking, and moods can become barriers to completing lifestyle interventions and managing obesity [30]. Furthermore, research has shown that experiencing depressive symptoms, which may result from prolonged focus on negative thinking and feelings, can contribute to dropout in weight-loss trials [33].

Along with the aforementioned psychological attributes, some studies have found that males seem to discontinue studies more often than females [30,34,35], and that older people tend to drop out of studies earlier than younger people [30]. Lower educational background has also been shown to contribute to dropout from obesity management studies, although there are also results that conflict with this [30]. The above-mentioned demographic factors, as well as personality and well-being related factors, have been addressed in examinations of attrition in previous longitudinal lifestyle interventions [36]. Regarding technology-based interventions, more evidence concerning the role of individual factors in committing to interventions is needed [14]. This research should target subgroups, in particular those with a severe risk of disease, such as people with overweight and obesity [30].

The present study examined the potential contribution of personality characteristics and psychological well-being to attrition within the technology-supported lifestyle intervention Motivation Makes the Move! (MoMaMo!) that was designed to reduce overweight and obesity and subsequent negative health outcomes. Based on evidence derived from previous weight management interventions, we hypothesized that scoring high in agreeableness, extraversion, and conscientiousness would reduce the risk of attrition in this technology-supported intervention. Furthermore, we hypothesized that persons who have experienced challenges with their psychological well-being would have a higher risk of dropping out from the study. Along with examining these hypotheses, we also test whether the potential findings are robust after controlling for participants’ age, sex, study group (personalized intervention vs general guidelines), and educational status. Since we consider that program adherence (in contrast with attrition) is necessary to achieve successful outcomes in interventions, we consider this study as a highly important starting point for the MoMaMo! project. This study strives to provide evidence on whether personality characteristics and psychological well-being measured at baseline should be taken under consideration in future technology-assisted lifestyle interventions to support participants’ commitment to the completion of such interventions.

## Methods

### Design of the Study

MoMaMo! was a part of the Bits of Health program, supported by Business Finland, the national governmental agency to support technological development and innovation [37]. MoMaMo! was registered at ClinicalTrials.gov (protocol record TYH2016215, NCT02686502). Recruitment for the study started in April 2016 and the last follow-ups were performed in April 2020.

The overarching aim of the MoMaMo! study was to decrease and prevent sedentary lifestyles and obesity and subsequent health consequences among physically inactive and overweight or obese people. Furthermore, the study aimed to develop and validate lasting and individualized IT- and mHealth-assisted behavior change methodologies and practices for citizen engagement in health, well-being, and prevention of diseases. Specifically, the study aimed to quantify benefits and mechanisms of individualized exercise training in comparison with general guidelines. It also strived for identifying key elements to increase the adherence to and effectiveness of an individualized physical activity intervention and weight management program incorporating mHealth and other health technology.

The primary outcome measure in the MoMaMo! study was maximal oxygen uptake (VO_2max_). VO_2max_ is a key parameter of cardiorespiratory fitness and an independent risk factor for several noncommunicable diseases and symptoms; it is associated with health-related quality of life [4]. VO_2max_ was assessed during a step-incremental cycle ergometer test (until volitional fatigue) with breath-by-breath alveolar gas exchange and pulmonary ventilation measurements. The highest 30-second moving average was calculated to obtain VO_2max_.

### Recruitment and Participants

**Figure 1:**
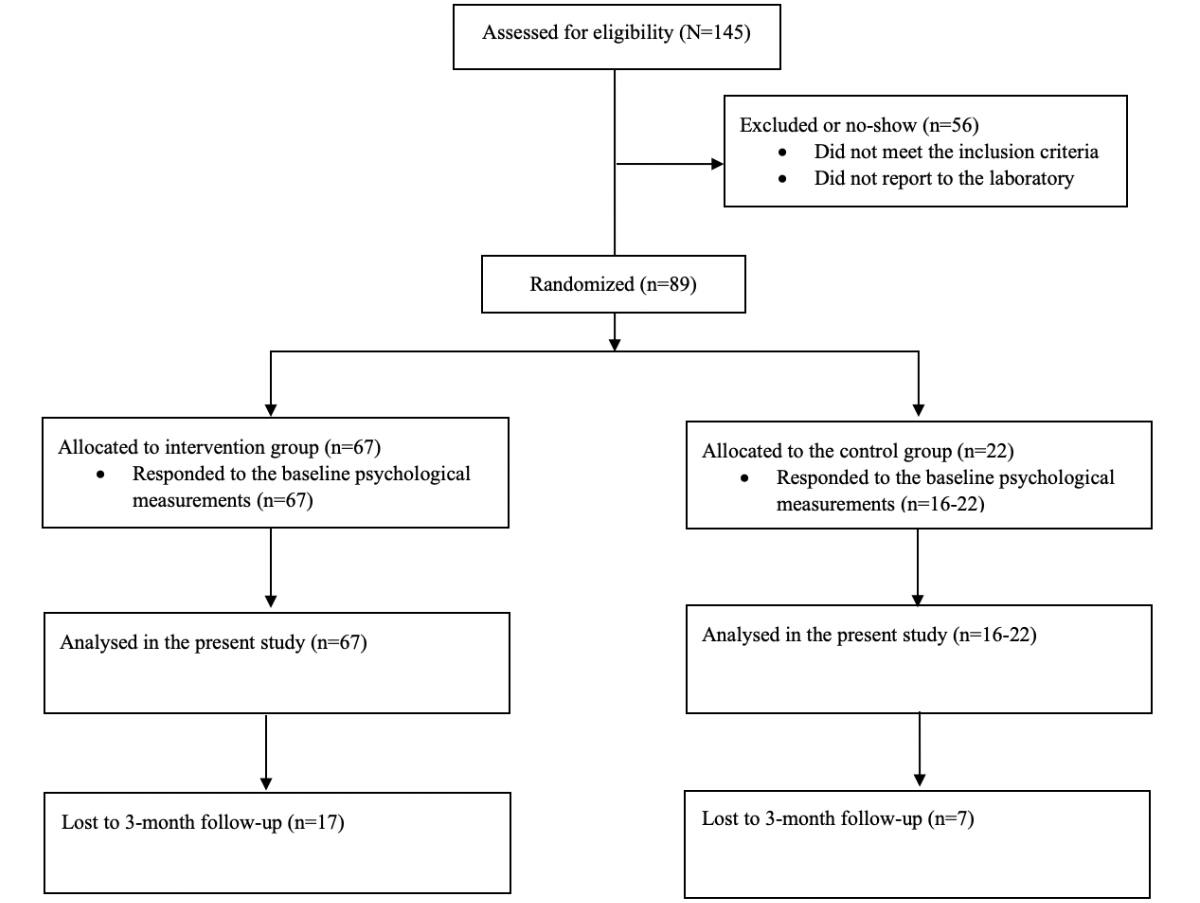
Flow diagram of the study (N=89).

Voluntary subjects were aged 18 to 40 years at entry, had BMI ≥ 27.5, had a referral from a physician for a consultation with a lifestyle clinic due to physical inactivity and overweight or obesity, and were deemed suitable for exercise testing and training. The subjects were recruited from different health care institutions in the Helsinki metropolitan area (Figure 1). In detail, participant recruitment was conducted by local public and private occupational health clinics by internet advertisement and recommendations by physicians. In addition, participants were recruited from the local University of Applied Sciences by internet advertisements. Exclusion criteria included the presence of a neurological or psychiatric disorder, use of medication influencing glucose homeostasis (except insulin) or autonomic nervous system function (eg, β-blockers or selective serotonin reuptake inhibitors), pregnancy, physical disability, substance abuse, significant co-operation difficulties, smoking, and severe anemia. The initial sample consisted of 89 subjects, of which 34 (38%) were men and 55 (62%) were women.

### MoMaMo! Intervention

The participants (N=89) were randomized into 3 groups. The principal investigator of the study generated the random allocation sequence. Research nurses, who were in charge of scheduling laboratory visit times, randomly allocated the participants to groups after the participants agreed to voluntarily participate in the study. A blinded draw of a paper containing the numbers 1, 2a, or 2b was performed to randomly allocate participant to group 1 (the general guidelines group), 2a (the individualized intervention group), or 2b (the highly individualized intervention group).

Group 1 visited the laboratory 3 times. During the first visit, the participants’ height, weight, and body composition were assessed. The participants filled out self-report questionnaires on physical activity, psychology, and music use, as well as their work productivity and activity impairment. During the second visit, a physician examined each subject to ensure their suitability for exercise testing and training. Thereafter, each subject performed a step-incremental cardiopulmonary exercise test on a cycle ergometer (Monark Ergomedic 839 E, Monark Exercise AB) until voluntary fatigue. Data included recordings of pulmonary ventilation (Triple V, Jaeger Mijnhardt), alveolar gas exchange (Oxycon Pro), electrocardiography (ECG; PowerLab, AD Instruments) and the ratio of perceived exertion (RPE). On the third visit, blood tests were done, and the subjects received brief feedback on their results and measured health outcomes. Furthermore, group 1 subjects were provided general guidelines for a healthy diet and physical activity [38,39].

Groups 2a and 2b underwent the same measurements as group 1. Group 2a subjects also performed a submaximal step-incremental cycle ergometer test with ECG and RPE recordings during an additional visit. After completing all measurements, a personalized feedback meeting was organized for each subject in groups 2a and 2b. In this final meeting, each group 2a and group 2b subject received healthy lifestyle habit advice and an exercise prescription based on their results and their own preferences. For group 2a, a submaximal cycle ergometer test was used to personalize the exercise prescription. Thus, an estimate of maximal oxygen consumption (VO_2max_), work-rate specific heart rate, and RPE were used to personalize the volume and intensity of the exercise prescription. For group 2b, measured VO_2max_, determined ventilatory thresholds, heart rate response, and RPE during a maximal cycle ergometer test were used to provide a highly individualized exercise prescription. The purpose of using the simpler exercise test for the exercise prescriptions given to group 2a was to examine its usefulness in comparison with the more highly detailed cardiopulmonary exercise test given to group 2b.

To support the participants’ own planning and follow-up of their physical activity, exercise training, and other health behaviors, subjects in groups 2a and 2b were instructed to use smartphone apps. Sports Tracker (Amer Sports Digital Services Oy) with a heart rate belt (Suunto Oy) was used to guide and record exercise training, Argus (Azumio Inc) or its equivalent to count daily steps, Emotion Tracker (F8) to assess emotions, and Weight Diary (CurlyBrace Apps Ltd) or its equivalent to measure weight. The study web page provided further instructions and support for training. A 3-month Spotify gift card, along with example playlists, was provided to the subjects assigned to groups 2a and 2b to motivate their exercise training and support relaxation. The participants were instructed to fill out a web-based food diary (Nutri-Flow Oy) for ≥3 days at the beginning of intervention. The diary provided an analysis of nutritional habits, along with personalized feedback for suggested modifications. Subjects in groups 2a and 2b were also encouraged to report their training and weight loss on the study website (note the website was functional for the study duration and is presently not accessible) [40].

Sample size determination and power calculation (G*Power, Universität Kiel) were based on the expected increase in VO_2max_ (the primary outcome measure in the MoMaMo! study) after a 3-month exercise intervention in groups 1, 2a, and 2b. Our a priori hypothesis was that both the individualized (group 2a) and highly individualized (group 2b) interventions would induce a similar increase in VO_2max_ and be more beneficial than the intervention based on general guidelines (group 1). Therefore, groups 2a and 2b were considered as a single group (group 2) in comparisons with group 1. Based on the literature and our previous data, we assumed that there would be a similar initial VO_2max_ value of 25 (SD 2.5) mL/kg/min (representing a poor or very poor classification) in all groups [41,42]. Our hypothesis was that VO_2max_ would increase 15% in group 2 and 5% in group 1. To determine a priori differences between 2 independent means, a 2-tailed *t* test with α error probability = .05, power = 0.80, and effect size *d*=0.92, we needed 20 subjects in both groups. Due to an assumed attrition rate of 30% in the overweight or obese subjects and with a goal to also perform comparisons between groups 2a and 2b, we sought to recruit and randomize 33 subjects into each group, for a total of 99 subjects.

Final collected data came from 89 participants, including 22 (25%) participants in group 1, who received general guidelines on physical activity and nutrition, and 67 (75%) participants in group 2, who received combined individualized exercise and physical activity prescriptions, a food diary, and the health technology apps described above (Figure 1). The study procedure and its reporting followed the CONSORT (Consolidated Standards of Reporting Trials of Electronic and Mobile Health Applications and Online Telehealth) statement [43].

### Psychological Questionnaires

Participants’ personality characteristics were assessed with the Short Five (S5) personality assessment [21]. This measure is designed to assess personality traits, including neuroticism, extraversion, openness to experience, agreeableness, and conscientiousness. Each dimension consists of 6 items, with higher scores reflecting higher levels of each trait. The scale ranges from –3 (the description is completely wrong/false) to 3 (the description is completely right or true). The sum scores of these 6 items were calculated. The reliability estimates (Cronbach α) were α=.84 for neuroticism, α=.75 for extraversion, α=.55 for openness to experience, α=.72 for agreeableness, and α=.65 for conscientiousness, indicating acceptable internal consistency for the items within each dimension. The major advantage of the S5 is that it allows for a detailed description of personality traits with a relatively small number of items compared to other instruments that assess the same components of personality [21]. This instrument has previously demonstrated good validity within general adult populations and correlates well with other measures of personality, including the NEO-Five-Factor Inventory [21].

Participants’ psychological well-being was studied with the RAND 36-item health survey, consisting of a set of quality-of-life measures. This measure is designed to capture the following health concepts: physical functioning, bodily pain, role limitations due to physical health challenges, role limitations due to emotional problems, emotional well-being, social functioning, vitality (energy and fatigue), and perceptions of general health [44]. Higher scores within the scale reflect a more favorable health state. In the present study, the reliability of the subscales ranged from good to excellent (physical functioning: α=.72; role limitations due to physical health challenges: α=.68; role limitations due to personal or emotion-related problems: α=.78; vitality: α=.78; emotional well-being: α=.79; social functioning: α=.91; bodily pain: α=.76; and general health perceptions: α=.73). In previous studies, this instrument has been demonstrated to be valid in general adult populations [31,44].

Participants’ mood was examined using the Positive and Negative Affect Schedule (PANAS). PANAS is a 20-item self-reported measure consisting of two 10-item scales, one for positive affect and one for negative affect. Participants were requested to rate single-word items describing positive or negative emotions. Higher scores reflected higher intensity of an emotion (1 indicated “not at all” and 5 indicated “extremely”). One missing value within each scale was allowed when calculating the scores. PANAS has been regarded as reliable and valid in previous studies [45]. Within the present study, Cronbach α for the scale assessing positive affectivity was α=.83, and the corresponding value for the scale for negative affectivity was α=.86.

Participants’ depressive symptoms were assessed using the Beck Depression Inventory II (BDI-II) [46]. The BDI-II assesses 21 symptoms with a severity range from 0 (no symptoms) to 3 (severe level of depressive symptoms). A sum score of all items was computed for each participant (Table 1 shows descriptive statistics); no missing items were allowed. The reliability estimate (Cronbach α) for the depressive symptom scores was α=.90. The BDI-II has been demonstrated to be a valid instrument [46-48] and is an acknowledged standard in the measurement of depressive mood [46-48]. This instrument has also been demonstrated to be a useful screening tool for future risk for depression [46,47]. Each study group completed the above-mentioned self-administered questionnaires.

**Table 1:** Frequencies, percentages, and attrition rates of study variables.

Variables			Participants at month 0, n		Participants at month 3, n (%)		Attrition rate, n (%)
**Study group**							
	Group 1	22		15 (68)		7 (32)	
	Group 2	67		50 (75)		17 (25)	
	Total	89		65 (73)		24 (27)	
**Sex**							
	Men	34		27 (79)		7 (21)	
	Women	55		38 (69)		17 (31)	
	Total	89		65 (73)		24 (27)	
**Educational background**							
	Vocational school	24		11 (46)		13 (54)	
	Lower academic degree	35		29 (83)		6 (17)	
	Academic degree	27		24 (89)		3 (11)	
	Total	86		64 (74)		22 (26)	
**Psychological measures**							
	Personality	88		64 (73)		24 (27)	
	RAND-36	89		65 (73)		24 (27)	
	Positive and Negative Affect Schedule	89		65 (73)		24 (27)	
	Beck Depression Inventory II	83		61 (73)		22 (27)	

### Statistical Analyses

Statistical analyses focused on assessing attrition with respect to the participants’ self-reported personality characteristics and psychological well-being at the 3-month follow-up. The analyses were conducted using SPSS versions 25 and 27 (IBM Corp). Descriptive statistics of the data are presented as frequencies, percentages, means, and standard deviations in Tables 1-4. The minimum and maximum values are also reported in Tables 2-4.

**Table 2:** Mean values of personality characteristics in continuing (n=64) and dropout (n=24) groups of participants at the 3-month follow-up.

Variables	Continuing group (n=64)			Dropout group (n=24)	
	Minimum-maximum	Mean (SD)	Minimum-maximum		Mean (SD)
Neuroticism	–29.00 to 19.00	–8.28 (12.65)	–33.00 to 18.00		–6.13 (14.39)
Extraversion	19.00 to 30.00	7.31 (11.63)	–14.00 to 26.00		8.75 (11.40)
Openness	–2.00 to 35.00	14.34 (8.42)	–12.00 to 27.00		11.04 (9.56)
Agreeableness	–13.00 to 32.00	16.13 (10.17)	–7.00 to 32.00		14.92 (8.94)
Conscientiousness	–4.00 to 33.00	15.98 (9.02)	–8.00 to 33.00		15.25 (10.17)

**Table 3:** Mean values of psychological well-being indicators in continuing (n=61-65) and dropout (n=22-24) groups at the 3-month follow-up.

Variables	Continuing group (n=61-65)		Dropout group (n=22-24)	
	Minimum-maximum	Mean (SD)	Minimum-maximum	Mean (SD)
Physical functioning	50.00-100.00	91.23 (8.66)	60.00-100.00	91.25 (11.06)
Role limitations (physical)	0.00-100.00	89.62 (22.49)	50.00-100.00	93.75 (15.20)
Role limitations (psychological)	0.00-100.00	82.05 (31.22)	0.00-100.00	75.00 (37.11)
Vitality (energy/fatigue)	20.00-100.00	59.38 (16.83)	20.00-85.00	56.88 (21.51)
Emotional well-being	36.00-100.00	75.22 (13.68)	44.00-96.00	73.33 (15.72)
Social functioning	25.00-100.00	86.15 (16.55)	37.50-100.00	84.90 (21.17)
Bodily pain	22.50-100.00	78.35 (19.32)	32.50-100.00	74.38 (17.39)
General health perceptions	16.67-87.50	55.26 (16.49)	25.00-87.50	59.72 (17.71)
Positive mood	16.00-44.00	30.46(6.01)	21.00-40.00	30.29 (5.47)
Negative mood	10.00-30.00	13.74 (4.47)	10.00-26.00	14.46 (4.97)
Symptoms of depression	0.00-28.00	7.49 (6.37)	0.00-29.00	7.55 (7.32)

**Table 4:** Mean values of psychological well-being indicators with square root transformations in continuing (n=61-65) and dropout (n=22-24) groups at the 3-month follow-up.

Variables	Continuing group (n=61-65)		Dropout group (n=22-24)	
	Minimum-maximum	Mean (SD)	Minimum-maximum	Mean (SD)
Physical functioning	1.00-7.14	2.82 (1.37)	1.00-6.40	2.66 (1.66)
Role limitations (physical)	1.00-10.05	2.28 (2.50)	1.00-7.14	1.85 (2.00)
Symptoms of depression	0.00-5.29	2.47 (1.20)	0.00-5.39	2.36 (1.44)

The attrition analyses were conducted using hierarchical logistic regressions. Traditional statistical analysis methods have been used in previous longitudinal lifestyle interventions when examining attrition-related questions [36]. In the present study, the associations between the main predictors and the outcome, in other words, whether personality characteristics and indicators of psychological well-being contributed to attrition at the 3-month follow-up, were studied first (Table 5). Furthermore, the analyses were adjusted for the participants’ sex, age, study group (group 1 vs group 2), and educational status (1 indicating vocational school or high school, 2 indicating a lower academic degree, and 3 indicating a higher academic degree) to test the robustness of the results (Table 6). The potential confounding factors were added to the models in the first step, and the main predictors in the second step (Table 6). The predictive power (Nagelkerke *R*^2^) of the models is reported in Tables 5 and 6. For detailed estimates regarding the confounding factors, see Multimedia Appendix 1 (Tables S1-S16).

**Table 5:** Personality characteristics and indicators of psychological well-being predicting attrition at the 3-month follow-up.

Variables			B	SE		*P* value		OR		95% CI		Nagelkerke *R*^2^
**Personality characteristics**												
	Neuroticism	0.01		0.02	.49		1.01		0.98-1.05		0.01	
	Extraversion	0.01		0.02	.60		1.01		0.97-1.05		0.01	
	Openness	–0.04		0.03	.12		0.96		0.91-1.01		0.04	
	Agreeableness	–0.01		0.02	.61		0.99		0.94-1.04		0.00	
	Conscientiousness	–0.01		0.03	.74		0.99		0.94-1.04		0.00	
**Indicators of psychological well-being^a^**												
	Physical functioning	–0.07		0.17	.67		0.93		0.67-1.29		0.00	
	Role limitations (physical)	–0.09		0.11	.45		0.92		0.74-1.15		0.01	
	Role limitations (psychological)	–0.01		0.01	.37		0.99		0.98-1.01		0.01	
	Vitality (energy/fatigue)	–0.01		0.01	.56		0.99		0.97-1.02		0.01	
	Emotional well-being	–0.01		0.02	.58		0.99		0.96-1.02		0.01	
	Social functioning	–0.004		0.01	.77		1.00		0.97-1.02		0.00	
	Bodily pain	–0.01		0.01	.38		0.99		0.97-1.01		0.01	
	General health perceptions	0.02		0.02	.27		1.02		0.99-1.05		0.02	
	Positive mood	–0.01		0.04	.90		1.00		0.92-1.08		0.00	
	Negative mood	0.03		0.05	.51		1.03		0.94-1.14		0.01	
	Symptoms of depression	–0.07		0.20	.73		0.93		0.63-1.38		0.00	

^a^Square root transformations were applied to predictors of physical functioning, role limitations (physical), and symptoms of depression.

**Table 6:** Personality characteristics and psychological well-being predicting attrition at the 3-month follow-up after adjusting for covariates.

Variables			B		SE	*P* value			OR		95% CI		Nagelkerke *R*^2^
**Personality characteristics**													
	Neuroticism	–0.03		0.03			.26	0.97		0.92-1.02		0.27	
	Extraversion	0.03		0.03			.26	1.03		0.98-1.09		0.28	
	Openness	0.02		0.03			.46	0.98		0.92-1.04		0.27	
	Agreeableness	–0.02		0.03			.53	0.98		0.93-1.04		0.27	
	Conscientiousness	0.02		0.03			.50	1.02		0.96-1.09		0.27	
**Indicators of psychological health^a^**													
	Physical functioning	–0.13		0.19			.50	0.88		0.61-1.27		0.27	
	Role limitations (physical)	–0.12		0.12			.35	0.89		0.70-1.13		0.28	
	Role limitations (psychological)	–0.003		0.01			.71	1.00		0.98-1.01		0.27	
	Vitality (energy/fatigue)	0.00		0.02			.95	1.00		0.97-1.03		0.27	
	Emotional well-being	0.01		0.02			.76	1.01		0.97-1.05		0.27	
	Social functioning	0.00		0.02			.93	1.00		0.97-1.04		0.27	
	Bodily pain	–0.001		0.01			.97	1.00		0.97-1.03		0.27	
	General health perceptions	0.03		0.02			.08	1.03		1.00-1.07		0.31	
	Positive mood	0.06		0.05			.29	1.06		0.95-1.18		0.28	
	Negative mood	0.01		0.06			.99	1.00		0.88-1.13		0.27	
	Symptoms of depression	–0.38		0.25			.13	0.68		0.42-1.12		0.31	

^a^Square root transformations were applied to predictors of physical functioning, role limitations (physical), and symptoms of depression.

### Ethics Approval

Subjects gave their written informed consent prior to participating in the study. The study conformed to the Declaration of Helsinki and was approved by the Ethics Committee (approval number 384/13/03/00/2015) of the Hospital District of Helsinki and Uusimaa, Helsinki, Finland.

## Results

Of the 89 participants who were allocated to the intervention group (groups 2a and 2b, together comprising group 2), 65 (73%) participated in the 3-month follow-up. Tables 1-4 show further information on attrition rates and study variables. When the distributions of the variables were viewed within the whole sample, the distributions of personality factors were approximately symmetric. Regarding the psychological well-being indicators, the variable reflecting physical functioning was negatively skewed (skewness –1.75, kurtosis 4.47), as was the variable reflecting role limitations due to physical health challenges (skewness –2.49, kurtosis 5.95). The variable reflecting depressive symptoms was positively skewed (skewness 1.41, kurtosis 2.04). Skewness and kurtosis values exceeding the absolute value of 2 were considered as indicating severe nonnormality, and square root transformations were performed for these variables (Table 4). Considering other variables in RAND-36 and PANAS, no severe skewness was detected. A total of 77 of the 83 participants (93%) experienced only a minimal or small number of depressive symptoms (original scores 0-18), while 6 (7%) of the 83 participants experienced a moderate number of depressive symptoms (original scores 19-29). No information on severe depressive symptoms (original scores >30) was detected.

The examined personality characteristics and indicators of psychological well-being did not predict the risk for dropping out from the study at the 3-month follow-up (all *P* values >.05) (Table 5). The results remained the same when potential confounding factors, including age, sex, study group, and educational status, were adjusted for in the analyses (all *P* values >.05; Table 6). Perceptions of general health approached significance; in other words, health perceptions marginally predicted dropout from the study after covariate controls (*P*=.08; Table 6). Estimates for each confounder can be found in Multimedia Appendix 1 (Tables S1-S16).

## Discussion

### Principal Findings

The health-technology assisted MoMaMo! study focused on physically inactive overweight and obese men and women who were at risk for a permanent physically inactive lifestyle and lifestyle-related chronic diseases. We focused on assessing whether participant personality characteristics and indicators of psychological well-being contributed to attrition within the MoMaMo! study and whether these factors should be taken into consideration more carefully in future interventions to support adults’ commitment to technology-based intervention programs.

Our results showed that 65 (73%) of the 89 initially recruited participants were still participating at the 3-month follow-up. Similar, and also lower, adherence rates have been demonstrated in other longitudinal weight management interventions [27,49]. Specifically, participant attrition rates from eHealth interventions have previously been shown to exceed 20% [50]. Here, the examined personality dimensions of neuroticism, extraversion, openness to experience, agreeableness, and conscientiousness were not associated with the risk of dropping out from the study after the 3-month follow-up. Furthermore, the indicators of psychological well-being were not associated with the risk of attrition. All the results remained nonsignificant after adjusting for participant age, sex, study group, and educational status.

At present, studies on attrition in technology-based exercise intervention contexts are limited. Based on previous research [27], we carefully draw a hypothesis that scoring high in agreeableness and extraversion might reduce the risk of attrition. Our findings did not align with the evidence found by De Panfilis and colleagues [27] that characteristics related to agreeableness and extraversion (ie, reward dependence) may reduce the risk of dropping out in behavioral weight-loss interventions. Our study, however, is in the same direction as De Panfilis and colleague’s [27] conclusion that personality as a whole seems to not be involved in whether subjects attend interventions associated with behavioral change (eg, weight loss). Some previous evidence has also indicated that scoring low in novelty seeking, which has been shown to correlate with conscientiousness [23], is associated with the completion of longitudinal weight management interventions [28]. Based on this, we expected that scoring high in conscientiousness might be associated with study adherence in MoMaMo! However, this characteristic was not related to the outcome. One possibility for our study’s differences in relation to previous results could be that certain personality dimensions might be associated with later dropout [27]; in other words, personality-related dropout may occur after a longer period than 3 months. It has also been speculated that people who voluntarily join lifestyle programs have optimistic expectations for the program, and therefore their personality differences may not contribute to dropout at the beginning of the intervention [28]. Additionally, it has been suggested that there could be differences in lean and obese persons’ personality characteristics, making the results from different samples vary [28]. In conclusion, our results suggest that commitment to the present technology-assisted physical activity intervention at the 3-month follow-up was not linked to any specific personality characteristics in the overweight adults.

Previous studies have indicated that a lower level of health-related quality of life (eg, experiences of health and physical limitations), negative thoughts and moods, and depressive symptoms could contribute to participant attrition [30,33]. Participants’ expectations and emotions regarding technology-based interventions may also contribute to attrition [51]. Based on this evidence, it seems that people’s personal resources related to well-being might be a key element in completing projects such as lifestyle interventions. In the present study, participants scored high in dimensions reflecting positive affect or mood. Positive mood has previously been shown to predict adherence to interventions [30]. Furthermore, almost all participants (77/83, 93%) experienced only minimal or mild depressive symptoms, and no results on severe depressive symptoms were found. This might be the reason our results differed from those of Goode et al [33], who suggested that experiencing depression contributes to attrition. Taken together, we did not gain evidence for the contribution of the examined indicators of psychological well-being to dropout in the present study.

In addition to the above speculation, it is also possible that the designed intervention features in the technology-supported MoMaMo! trial were, in their current from, suitable for the recruited participants’ characteristics, so that dropout could not be attributed to their personality or levels of psychological well-being. Our findings suggest that the psychological qualities do not contribute to discontinuation of the technology-based lifestyle intervention program among overweight adults, and that tailoring interventions more carefully to personality and well-being levels may not be needed in supporting participants’ adherence to technology-assisted interventions during a 3-month period.

Potential causes of the relatively high attrition rate (27%) in our study remain, however, obscure. It has been stated that motivating users for behavior change before guiding them through action planning [35] could support their adherence to lifestyle interventions. The role of social support, such as integrating face-to-face intervention delivery strategies to technology-based intervention programs, may be a contributor to participants’ motivation to commit to these studies [52]. Individuals may also be more likely to drop out of trials if they do not meet weight loss goals [53]. Use of positive reinforcement may also be beneficial, especially in cases where reaching outcomes requires time (eg, losing weight) [51]. Based on these perspectives, it seems possible that more attention should be paid to motivational factors, referring to the inner states that energize and direct behaviors. At present, examination of the effectiveness of the MoMaMo! study is ongoing, and future studies on attrition could focus on assessing motivational elements beyond the present research, such as on participants’ meeting intervention goals.

### Limitations

This study focused on assessing the potential role of personality and psychological well-being in study attrition. Other factors, such as physical or social circumstances (eg, sudden outbreaks of disease or lack of social support) and those related to meeting goals were not assessed. Furthermore, specific study variables, including psychological well-being, were studied only in the beginning of the intervention. As psychological well-being, along with many other factors relating to human experience, fluctuates over time, it will be important to address the dynamic nature of this phenomenon in relation to study attrition (eg, using survival analyses) in future studies. Additionally, we cannot be absolutely certain whether the participants who did not come to the laboratory follow-up tests quit only the study or also their intervention program at the individual level. That is, they may or may not have continued changing or improving their lifestyles after they discontinued their participation in the MoMaMo! study. Although all the instruments’ subscales were considered acceptable according to general rules, openness to experience had poor reliability (Cronbach α=.55). Some studies have demonstrated similar α values when optimal subset items reflecting personality dimensions were researched [54]. Generally, the α coefficient reliability has been high in this instrument’s validation studies [21]. Furthermore, due to the relatively high attrition rate, some of the analyses performed in the study may lack statistical power. Consequently, these study results can be regarded only as directional. Studies with larger sample sizes and longer follow-up periods are needed to confirm our results.

### Strengths

We were able to study attrition with a well-validated set of psychological questionnaires. To our knowledge, this is one of the first studies assessing attrition in technology-supported, longitudinal lifestyle interventions conducted in overweight or obese adults. Our study is also one of the first to provide information on the role of participant psychological profiles in technology-supported interventions that aim at increasing physical activity and VO_2max_.

### Conclusions

The personality characteristics and indicators of psychological well-being examined in this study did not contribute to participant attrition in the technology-supported MoMaMo! lifestyle intervention conducted in overweight adults. As attrition remains a challenge within longitudinal, technology-supported lifestyle interventions, attention should be paid to the potentially dynamic nature of personality and psychological well-being, as well as to elements beyond these.

## Appendix

Multimedia Appendix 1 Detailed estimates for the confounding factors.

Multimedia Appendix 2 CONSORT-eHEALTH checklist (V 1.6.2).

## Abbreviations

BDI-II: Beck Depression Inventory II
CONSORT: Consolidated Standards of Reporting Trials of Electronic and Mobile Health Applications and Online Telehealth
ECG: electrocardiography
mHealth: mobile health
MoMaMo!: Motivation Makes the Move!
PANAS: Positive and Negative Affect Schedule
RPE: ratio of perceived exertion
S5: Short Five
VO_2max_: maximal oxygen uptake
