# Supplementary material for: Identifying Personality Characteristics and Indicators of Psychological Well-Being Associated With Attrition in the Motivation Makes the Move! Physical Activity Intervention: Randomized Technology-Supported Trial
Source: JMIR Form Res. 2022 Nov 25;6(11):e30285. doi: 10.2196/30285 (PMC9736762; doi:10.2196/30285)
Supplement: Multimedia Appendix 1 [file formative_v6i11e30285_app1.docx]

**Table S1**. Neuroticism predicting the attrition in the 3-month follow up adjusting for covariates^1,2^.

|  | B | SE | *P* value | OR | 95%CI  (upper) | 95%CI  (lower) |
| --- | --- | --- | --- | --- | --- | --- |
| Age | -.04 | .05 | .46 | .96 | .87 | 1.07 |
| Sex (1) | .34 | .62 | .59 | 1.40 | .42 | 4.70 |
| Study group (1) | -1.41 | .72 | .05 | .24 | .06 | 1.00 |
| Education |  |  | .00 |  |  |  |
| Education (1) | -2.08 | .70 | .00 | .13 | .03 | .49 |
| Education (2) | -2.83 | .92 | .00 | .06 | .01 | .36 |
| Neuroticism | -.03 | .03 | .26 | .97 | .92 | 1.02 |

^1^Age. sex. study group and education were added to the model in the first step. Neuroticism was added in the

second step

^2^The variable’s first category was defined as a reference category

**Table S2**. Extraversion predicting the attrition in the 3-month follow up adjusting for covariates^1,2^.

|  | B | SE | *P* value | OR | 95%CI  (upper) | 95%CI  (lower) |
| --- | --- | --- | --- | --- | --- | --- |
| Age | -.02 | .05 | .72 | .98 | .89 | 1.08 |
| Sex (1) | .18 | .60 | .76 | 1.20 | .37 | 3.85 |
| Study group (1) | -1.31 | .69 | .06 | .27 | .07 | 1.05 |
| Education |  |  | .00 |  |  |  |
| Education (1) | -2.04 | .70 | .00 | .13 | .03 | .51 |
| Education (2) | -2.64 | .86 | .00 | .07 | .01 | .39 |
| Extraversion | .03 | .03 | .26 | 1.03 | .98 | 1.09 |

^1^Age. sex. study group and education were added to the model in the first step. Extraversion was added in the

second step

^2^The variable’s first category was defined as a reference category

**Table S3**. Openness to experience predicting the attrition in the 3-month follow up adjusting for covariates^1,2^.

|  | B | SE | *P* value | OR | 95%CI  (upper) | 95%CI  (lower) |
| --- | --- | --- | --- | --- | --- | --- |
| Age | -.02 | .05 | .70 | .98 | .89 | 1.08 |
| Sex (1) | .15 | .59 | .80 | 1.16 | .37 | 3.68 |
| Study group (1) | -1.17 | .68 | .08 | .31 | .08 | 1.16 |
| Education |  |  | .01 |  |  |  |
| Education (1) | -1.91 | .69 | .01 | .15 | .04 | .57 |
| Education (2) | -2.41 | .83 | .00 | .09 | .02 | .46 |
| Openness | -.02 | .03 | .46 | .98 | .92 | 1.04 |

^1^Age. sex. study group and education were added to the model in the first step. Openness to experience was added in the

second step

^2^The variable’s first category was defined as a reference category

**Table S4**. Agreeableness predicting the attrition in the 3-month follow up adjusting for covariates^1,2^.

|  | B | SE | *P* value | OR | 95%CI  (upper) | 95%CI  (lower) |
| --- | --- | --- | --- | --- | --- | --- |
| Age | -.01 | .05 | .78 | .99 | .89 | 1.09 |
| Sex (1) | .25 | .61 | .69 | 1.28 | .39 | 4.27 |
| Study group (1) | -1.14 | .68 | .09 | .32 | .08 | 1.21 |
| Education |  |  | .00 |  |  |  |
| Education (1) | -1.93 | .69 | .01 | .15 | .04 | .56 |
| Education (2) | -2.46 | .83 | .00 | .09 | .02 | .44 |
| Agreeableness | -.02 | .03 | .53 | .98 | .93 | 1.04 |

^1^Age. sex. study group and education were added to the model in the first step. Agreeableness was added in the

second step

^2^The variable’s first category was defined as a reference category

**Table S5**. Conscientiousness predicting the attrition in the 3-month follow up adjusting for covariates^1,2^.

|  | B | SE | *P* value | OR | 95%CI  (upper) | 95%CI  (lower) |
| --- | --- | --- | --- | --- | --- | --- |
| Age | -.03 | .05 | .62 | .98 | .88 | 1.08 |
| Sex (1) | .15 | .59 | .80 | 1.16 | .37 | 3.67 |
| Study group (1) | -1.28 | .69 | .07 | .28 | .07 | 1.08 |
| Education |  |  | .00 |  |  |  |
| Education (1) | -2.06 | .71 | .00 | .13 | .03 | .51 |
| Education (2) | -2.55 | .85 | .00 | .08 | .02 | .41 |
| Conscientiousness | .02 | .03 | .50 | 1.02 | .96 | 1.09 |

^1^Age. sex. study group and education were added to the model in the first step. Conscientiousness was added in the

second step

^2^The variable’s first category was defined as a reference category

**Table S6**. Physical functioning predicting the attrition in the 3-month follow up adjusting for covariates^1,2^.

|  | B | SE | *P* value | OR | 95%CI  (upper) | 95%CI  (lower) |
| --- | --- | --- | --- | --- | --- | --- |
| Age | -.02 | .05 | .63 | .98 | .88 | 1.08 |
| Sex (1) | .19 | .59 | .75 | 1.21 | .38 | 3.81 |
| Study group (1) | -1.15 | .68 | .09 | .32 | .08 | 1.19 |
| Education |  |  | .00 |  |  |  |
| Education (1) | -1.98 | .69 | .00 | .14 | .04 | .53 |
| Education (2) | -2.55 | .83 | .00 | .08 | .02 | .40 |
| Physical functioning | -.13 | .19 | .50 | .88 | .61 | 1.27 |

^1^Age. sex. study group and education were added to the model in the first step. Physical functioning was added in the

second step

^2^The variable’s first category was defined as a reference category

**Table S7**. Role limitations (physical) predicting the attrition in the 3-month follow up adjusting

for covariates^1,2^.

|  | B | SE | *P* value | OR | 95%CI  (upper) | 95%CI  (lower) |
| --- | --- | --- | --- | --- | --- | --- |
| Age | -.03 | .05 | .58 | .97 | .88 | 1.08 |
| Sex (1) | .16 | .59 | .79 | 1.18 | .37 | 3.73 |
| Study group (1) | -1.26 | .69 | .07 | .28 | .07 | 1.09 |
| Education |  |  | .00 |  |  |  |
| Education (1) | -1.97 | .69 | .00 | .14 | .04 | .54 |
| Education (2) | -2.50 | .84 | .00 | .08 | .02 | .42 |
| Role limitations (phys.) | -.12 | .12 | .35 | .89 | .70 | 1.13 |

^1^Age. sex. study group and education were added to the model in the first step. Role limitations (physical) was added in the second step

^2^The variable’s first category was defined as a reference category

**Table S8**. Role limitations (psychological) predicting the attrition in the 3-month follow up

adjusting for covariates^1,2^.

|  | B | SE | *P* value | OR | 95%CI  (upper) | 95%CI  (lower) |
| --- | --- | --- | --- | --- | --- | --- |
| Age | -.02 | .05 | .71 | .98 | .89 | 1.08 |
| Sex (1) | .20 | .59 | .73 | 1.23 | .39 | 3.88 |
| Study group (1) | -1.09 | .69 | .11 | .34 | .09 | 1.30 |
| Education |  |  | .00 |  |  |  |
| Education (1) | -1.92 | .69 | .01 | .15 | .04 | .57 |
| Education (2) | -2.48 | .84 | .00 | .08 | .02 | .43 |
| Role limitations (psych.) | -.00 | .01 | .71 | 1.00 | .98 | 1.01 |

^1^Age. sex. study group and education were added to the model in the first step. Role limitations (psychological) was added in the second

step

^2^The variable’s first category was defined as a reference category

**Table S9**. Vitality predicting the attrition in the 3-month follow up adjusting

for covariates^1,2^.

|  | B | SE | *P* value | OR | 95%CI  (upper) | 95%CI  (lower) |
| --- | --- | --- | --- | --- | --- | --- |
| Age | -.02 | .05 | .67 | .98 | .89 | 1.08 |
| Sex (1) | .18 | .59 | .76 | 1.20 | .38 | 3.77 |
| Study group (1) | -1.16 | .69 | .09 | .31 | .08 | 1.21 |
| Education |  |  | .00 |  |  |  |
| Education (1) | -1.94 | .69 | .01 | .14 | .04 | .55 |
| Education (2) | -2.52 | .86 | .00 | .08 | .02 | .44 |
| Vitality | .00 | .02 | .95 | 1.00 | .97 | 1.03 |

^1^Age. sex. study group and education were added to the model in the first step. Vitality was added in the second step

^2^The variable’s first category was defined as a reference category

**Table S10**. Emotional wellbeing predicting the attrition in the 3-month follow up adjusting

for covariates^1,2^.

|  | B | SE | *P* value | OR | 95%CI  (upper) | 95%CI  (lower) |
| --- | --- | --- | --- | --- | --- | --- |
| Age | -.02 | .05 | .66 | .98 | .89 | 1.08 |
| Sex (1) | .18 | .59 | .76 | 1.19 | .38 | 3.76 |
| Study group (1) | -1.21 | .70 | .08 | .30 | .08 | 1.17 |
| Education |  |  | .00 |  |  |  |
| Education (1) | -1.96 | .69 | .00 | .14 | .04 | .54 |
| Education (2) | -2.55 | .86 | .00 | .08 | .02 | .42 |
| Emotional wellbeing | .01 | .02 | .76 | 1.01 | .97 | 1.05 |

^1^Age. sex. study group and education were added to the model in the first step. Emotional wellbeing was added in the second step

^2^The variable’s first category was defined as a reference category

**Table S11**. Social functioning predicting the attrition in the 3-month follow up adjusting

for covariates^1,2^.

|  | B | SE | *P* value | OR | 95%CI  (upper) | 95%CI  (lower) |
| --- | --- | --- | --- | --- | --- | --- |
| Age | -.02 | .05 | .67 | .98 | .89 | 1.08 |
| Sex (1) | .19 | .59 | .75 | 1.20 | .38 | 3.80 |
| Study group (1) | -1.17 | .70 | .09 | .31 | .08 | 1.22 |
| Education |  |  | .00 |  |  |  |
| Education (1) | -1.94 | .68 | .01 | .14 | .04 | .55 |
| Education (2) | -2.51 | .84 | .00 | .08 | .02 | .42 |
| Social functioning | .00 | .02 | .93 | 1.00 | .97 | 1.04 |

^1^Age. sex. study group and education were added to the model in the first step. Social functioning was added in the second step

^2^The variable’s first category was defined as a reference category

**Table S12**. Bodily pain predicting the attrition in the 3-month follow up adjusting

for covariates^1,2^.

|  | B | SE | *P* value | OR | 95%CI  (upper) | 95%CI  (lower) |
| --- | --- | --- | --- | --- | --- | --- |
| Age | -.02 | .05 | .68 | .98 | .89 | 1.08 |
| Sex (1) | .17 | .60 | .77 | 1.19 | .37 | 3.88 |
| Study group (1) | -1.15 | .67 | .09 | .32 | .08 | 1.18 |
| Education |  |  | .00 |  |  |  |
| Education (1) | -1.94 | .69 | .01 | .14 | .04 | .55 |
| Education (2) | -2.50 | .85 | .00 | .08 | .02 | .43 |
| Bodily pain | -.00 | .01 | .97 | 1.00 | .97 | 1.03 |

^1^Age. sex. study group and education were added to the model in the first step. Bodily pain was added in the second step

^2^The variable’s first category was defined as a reference category

**Table S13**. General health perceptions predicting the attrition in the 3-month follow up

adjusting for covariates^1,2^.

|  | B | SE | *P* value | OR | 95%CI  (upper) | 95%CI  (lower) |
| --- | --- | --- | --- | --- | --- | --- |
| Age | -.01 | .05 | .84 | .99 | .90 | 1.10 |
| Sex (1) | .32 | .60 | .60 | 1.37 | .42 | 4.49 |
| Study group (1) | -1.18 | .69 | .09 | .31 | .08 | 1.18 |
| Education |  |  | .00 |  |  |  |
| Education (1) | -2.21 | .73 | .00 | .11 | .03 | .46 |
| Education (2) | -3.01 | .90 | <.001 | .05 | .01 | .29 |
| General health | .03 | .02 | .08 | 1.03 | 1.00 | 1.07 |

^1^Age. sex. study group and education were added to the model in the first step. General health perceptions was added in the second step

^2^The variable’s first category was defined as a reference category

**Table S14**. Positive mood predicting the attrition in the 3-month follow up

adjusting for covariates^1,2^.

|  | B | SE | *P* value | OR | 95%CI  (upper) | 95%CI  (lower) |
| --- | --- | --- | --- | --- | --- | --- |
| Age | -.02 | .05 | .63 | .98 | .88 | 1.08 |
| Sex (1) | .11 | .59 | .85 | 1.12 | .35 | 3.57 |
| Study group (1) | -1.21 | .69 | .08 | .30 | .08 | 1.15 |
| Education |  |  | .00 |  |  |  |
| Education (1) | -2.17 | .73 | .00 | .11 | .03 | .48 |
| Education (2) | -2.85 | .93 | .00 | .06 | .01 | .36 |
| Positive mood | .06 | .05 | .29 | 1.06 | .95 | 1.18 |

^1^Age. sex. study group and education were added to the model in the first step. Positive mood was added at the second step

^2^The variable’s first category was defined as a reference category

**Table S15**. Negative mood predicting the attrition in the 3-month follow up

adjusting for covariates^1,2^.

|  | B | SE | *P* value | OR | 95%CI  (upper) | 95%CI  (lower) |
| --- | --- | --- | --- | --- | --- | --- |
| Age | -.02 | .05 | .68 | .98 | .88 | 1.08 |
| Sex (1) | .18 | .59 | .76 | 1.20 | .38 | 3.79 |
| Study group (1) | -1.15 | .71 | .11 | .32 | .08 | 1.28 |
| Education |  |  | .00 |  |  |  |
| Education (1) | -1.94 | .69 | .01 | .14 | .04 | .55 |
| Education (2) | -2.50 | .84 | .00 | .08 | .02 | .42 |
| Negative mood | .00 | .06 | .99 | 1.00 | .88 | 1.13 |

^1^Age. sex. study group and education were added to the model in the first step. Negative mood was added in the second step

^2^The variable’s first category was defined as a reference category

**Table S16**. Symptoms of depression predicting the attrition in the 3-month follow up

adjusting for covariates^1,2^.

|  | B | SE | *P* value | OR | 95%CI  (upper) | 95%CI  (lower) |
| --- | --- | --- | --- | --- | --- | --- |
| Age | -.04 | .06 | .53 | .97 | .87 | 1.08 |
| Sex (1) | .22 | .62 | .72 | 1.25 | .37 | 4.24 |
| Study group (1) | -1.26 | .79 | .11 | .29 | .06 | 1.35 |
| Education |  |  | .00 |  |  |  |
| Education (1) | -2.44 | .77 | .00 | .09 | .02 | .40 |
| Education (2) | -2.51 | .87 | .00 | .08 | .02 | .45 |
| Depressive symptoms | -.38 | .25 | .13 | .68 | .42 | 1.12 |

^1^Age. sex. study group and education were added to the model in the first step. Depressive symptoms was added in the second step.

^2^The variable’s first category was defined as a reference category
