# Supplementary material for: Identifying Personality Characteristics and Indicators of Psychological Well-Being Associated With Attrition in the Motivation Makes the Move! Physical Activity Intervention: Randomized Technology-Supported Trial
Source: JMIR Form Res. 2022 Nov 25;6(11):e30285. doi: 10.2196/30285 (PMC9736762; doi:10.2196/30285)
Supplement: Multimedia Appendix 2 [file formative_v6i11e30285_app2.pdf]

CONSORT-EHEALTH Checklist V1.6.2 Report

Manuscript Number: 30285

(based on CONSORT-EHEALTH V1.6), available at [http://tinyurl.com/consort-ehealth-v1-6].

Date completed

11/18/2022 4:50:30

by

Juha Peltonen

Identifying Personality Characteristics and Indicators of Psychological Well-Being Associated With Attrition in the Motivation Makes the Move! Physical Activity Intervention:
Randomized Technology-Supported Trial

TITLE

1a-i) Identify the mode of delivery in the title

Yes, we stated that this trial is "Randomized Technology-Supported Trial" in the manuscript's title.

1a-ii) Non-web-based components or important co-interventions in title

1a-iii) Primary condition or target group in the title

Yes. The title of the manuscript is “Identifying Personality Characteristics and Indicators of Psychological Well-Being Associated With Attrition in the Motivation Makes the Move! Physical Activity Intervention: Randomized Technology-Supported Trial"

ABSTRACT

1b-i) Key features/functionalities/components of the intervention and comparator in the METHODS section of the ABSTRACT

Yes. The key features of the present study in the abstract’s method section. “Participants (N=89) were adults from the Motivation Makes the Move! intervention study. Data attrition was studied after a 3-month follow-up. Participants’ personality characteristics were studied using the Short Five self-report questionnaire. Psychological well-being indicators were assessed with the RAND 36-item health survey, Positive and Negative Affect Schedule, and Beck Depression Inventory. Logistic regression analyses were conducted to assess the risk of discontinuing the study. The analyses were adjusted for sex, age, study group, and educational status.”

1b-ii) Level of human involvement in the METHODS section of the ABSTRACT

1b-iii) Open vs. closed, web-based (self-assessment) vs. face-to-face assessments in the METHODS section of the ABSTRACT

1b-iv) RESULTS section in abstract must contain use data

1b-v) CONCLUSIONS/DISCUSSION in abstract for negative trials

INTRODUCTION

2a-i) Problem and the type of system/solution

Yes, the study’s scientific background and the rationale of the study have been presented in the manuscript’s introduction. See also manuscript’s page 3 “This study strives to provide evidence on whether personality characteristics and psychological well-being measured at baseline should be taken under consideration in future technology-assisted lifestyle interventions to support participants’ commitment to the completion of such interventions”.

2a-ii) Scientific background, rationale: What is known about the (type of) system

Yes. “The present study examined the potential contribution of personality characteristics and psychological well-being to attrition within the technology-supported lifestyle intervention Motivation Makes the Move! (MoMaMo!) that was designed to reduce overweight and obesity and subsequent negative health outcomes.” “Since we consider that program adherence (in contrast with attrition) is necessary to achieve successful outcomes in interventions, we consider this study as a highly important starting point for the MoMaMo! project. This study strives to provide evidence on whether personality characteristics and psychological well-being measured at baseline should be taken under consideration in future technology-assisted lifestyle interventions to support participants’ commitment to the completion of such interventions.”

Does your paper address CONSORT subitem 2b?

Yes, specific objectives and hypotheses have been presented in the manuscript. “Based on evidence derived from previous weight management interventions, we hypothesized that scoring high in agreeableness, extraversion, and conscientiousness would reduce the risk of attrition in this technology-supported intervention. Furthermore, we hypothesized that persons who have experienced challenges with their psychological well-being would have a higher risk of dropping out from the study. Along with examining these hypotheses, we also test whether the potential findings are robust after controlling for participants’ age, sex, study group (personalized intervention vs general guidelines), and educational status.”

METHODS

3a) CONSORT: Description of trial design (such as parallel, factorial) including allocation ratio

Yes, the description of the trial design has been provided in the manuscript’s Methods section. Please find pages 3-5. Please see the Figure 1 (Flow diagram of the study).

3b) CONSORT: Important changes to methods after trial commencement (such as eligibility criteria), with reasons

NA. No changes were made to methods after trial commencement.

3b-i) Bug fixes, Downtimes, Content Changes

4a) CONSORT: Eligibility criteria for participants

Yes, the description of the eligibility criteria for the participants has been provided in the manuscript’s Methods section. “Voluntary subjects were aged 18 to 40 years at entry, had BMI ≥ 27.5, had a referral from a physician for a consultation with a lifestyle clinic due to physical inactivity and overweight or obesity, and were deemed suitable for exercise testing and training.” “Exclusion criteria included the presence of a neurological or psychiatric disorder, use of medication influencing glucose homeostasis (except insulin) or autonomic nervous system function (e.g., β-blockers or selective serotonin reuptake inhibitors), pregnancy, physical disability, substance abuse, significant co-operation difficulties, smoking, and severe anemia.”

4a-i) Computer / Internet literacy

4a-ii) Open vs. closed, web-based vs. face-to-face assessments:

Yes, this has been described in the manuscript’s Methods section. “The subjects were recruited from different health care institutions in the Helsinki metropolitan area (Figure 1). In detail, participant recruitment was conducted by local public and private occupational health clinics by internet advertisement and recommendations by physicians. In addition, participants were recruited from the local University of Applied Sciences by internet advertisements.”

4a-iii) Information giving during recruitment

4b) CONSORT: Settings and locations where the data were collected

Yes, this has been addressed in the manuscript’s Methods section. “The subjects were recruited from different health care institutions in the Helsinki metropolitan area”. Please find Figure 1. All questionnaire and laboratory data collection took place at the Department of Sport and Exercise Medicine, Clinicum, University of Helsinki, Finland.

4b-i) Report if outcomes were (self-)assessed through online questionnaires

Primary outcome (cardiorespiratory fitness, VO2max) was assessed during incremental exercise test in the laboratory. Secondary outcomes were assessed with outcome-specific methods (questionnaires, laboratory assays). Attrition was assessed by subject’s no-show to the laboratory despite a possibility to a new appointment(s).

4b-ii) Report how institutional affiliations are displayed

5) CONSORT: Describe the interventions for each group with sufficient details to allow replication, including how and when they were actually administered

5-i) Mention names, credential, affiliations of the developers, sponsors, and owners

5-ii) Describe the history/development process

5-iii) Revisions and updating

5-iv) Quality assurance methods

5-v) Ensure replicability by publishing the source code, and/or providing screenshots/screen-capture video, and/or providing flowcharts of the algorithms used

**5-vi) Digital preservation**		
**5-vii) Access**		
Yes, this has been reported in the article's Methods section. The study web page (functional for the study duration) provided instructions and support for training to the participants. "Furthermore, group 1 subjects were provided general guidelines for a healthy diet and physical activity." "After completing all measurements, a personalized feedback meeting was organized for each subject in groups 2a and 2b. In this final meeting, each group 2a and group 2b subject received healthy lifestyle habit advice and an exercise prescription based on their results and their own preferences." To support the participants' own planning and follow-up of their physical activity, exercise training, and other health behaviors, subjects in groups 2a and 2b were instructed to use smartphone apps. Sports Tracker (Amer Sports Digital Services Oy) with a heart rate belt (Suunto Oy) was used to guide and record exercise training, Argus (Azumio Inc) or its equivalent to count daily steps, Emotion Tracker (F8) to assess emotions, and Weight Diary (CurlyBrace Apps Ltd) or its equivalent to measure weight. The study web page provided further instructions and support for training. A 3-month Spotify gift card, along with example playlists, was provided to the subjects assigned to groups 2a and 2b to motivate their exercise training and support relaxation. The participants were instructed to fill out a web-based food diary (Nutri-Flow Oy) for ≥3 days at the beginning of intervention. The diary provided an analysis of nutritional habits, along with personalized feedback for suggested modifications. Subjects in groups 2a and 2b were also encouraged to report their training and weight loss on the study website (note the website was functional for the study duration and is presently not accessible)."		
**5-viii) Mode of delivery, features/functionalities/components of the intervention and comparator, and the theoretical framework**		
Yes, the features of the intervention have been described in the article. Please see the Methods section and 5-vii above.		
**5-ix) Describe use parameters**		
**5-x) Clarify the level of human involvement**		
**5-xi) Report any prompts/reminders used**		
An email was sent to each participant at one-month mark after he/she had started the intervention to encourage asking questions (if any) on executing the intervention and/or using apps and/or the study website.		
**5-xii) Describe any co-interventions (incl. training/support)**		
NA. We did not include this type of information to the manuscript because no co-interventions were conducted.		
**6a) CONSORT: Completely defined pre-specified primary and secondary outcome measures, including how and when they were assessed**		
Yes, outcomes, including how and when they were assessed, are reported in the article's Methods section. "The primary outcome measure in the MoMaMo! study was maximal oxygen uptake (VO2max). VO2max is a key parameter of cardiorespiratory fitness and an independent risk factor for several noncommunicable diseases and symptoms; it is associated with health-related quality of life [4]. VO2max was assessed during a step-incremental cycle ergometer test (until volitional fatigue) with breath-by-breath alveolar gas exchange and pulmonary ventilation measurements. The highest 30-second moving average was calculated to obtain VO2max." For the present study, attrition at the 3-month mark and its association with personality was the key outcome. "The attrition analyses were conducted using hierarchical logistic regressions. Traditional statistical analysis methods have been used in previous longitudinal lifestyle interventions when examining attrition-related questions [36]. In the present study, the associations between the main predictors and the outcome, in other words, whether personality characteristics and indicators of psychological well-being contributed to attrition at the 3-month follow-up, were studied first (Table 5). Furthermore, the analyses were adjusted for the participants' sex, age, study group (group 1 vs group 2), and educational status (1 indicating vocational school or high school, 2 indicating a lower academic degree, and 3 indicating an academic degree) to test the robustness of the results (Table 6). The potential confounding factors were added to the models in the first step, and the main predictors in the second step (Table 6). The predictive power (Nagelkerke R2) of the models is reported in Tables 5 and 6. For detailed estimates regarding the confounding factors, see Multimedia Appendix 1 (Tables S1-S16)."		
**6a-i) Online questionnaires: describe if they were validated for online use and apply CHERRIES items to describe how the questionnaires were designed/deployed**		
**6a-ii) Describe whether and how "use" (including intensity of use/dosage) was defined/measured/monitored**		
**6a-iii) Describe whether, how, and when qualitative feedback from participants was obtained**		
**6b) CONSORT: Any changes to trial outcomes after the trial commenced, with reasons**		
Yes, this has been addressed in the manuscript's Methods section. "The subjects were recruited from different health care institutions in the Helsinki metropolitan area". Please find Figure 1. All questionnaire and laboratory data collection took place at the Department of Sport and Exercise Medicine, Clinicum, University of Helsinki, Finland.		
**7a) CONSORT: How sample size was determined**		
**7a-i) Describe whether and how expected attrition was taken into account when calculating the sample size**		
**7b) CONSORT: When applicable, explanation of any interim analyses and stopping guidelines**		
Yes, outcomes, including how and when they were assessed, are reported in the article's Methods section. "The primary outcome measure in the MoMaMo! study was maximal oxygen uptake (VO2max). VO2max is a key parameter of cardiorespiratory fitness and an independent risk factor for several noncommunicable diseases and symptoms; it is associated with health-related quality of life [4]. VO2max was assessed during a step-incremental cycle ergometer test (until volitional fatigue) with breath-by-breath alveolar gas exchange and pulmonary ventilation measurements. The highest 30-second moving average was calculated to obtain VO2max." For the present study, attrition at the 3-month mark and its association with personality was the key outcome. "The attrition analyses were conducted using hierarchical logistic regressions. Traditional statistical analysis methods have been used in previous longitudinal lifestyle interventions when examining attrition-related questions [36]. In the present study, the associations between the main predictors and the outcome, in other words, whether personality characteristics and indicators of psychological well-being contributed to attrition at the 3-month follow-up, were studied first (Table 5). Furthermore, the analyses were adjusted for the participants' sex, age, study group (group 1 vs group 2), and educational status (1 indicating vocational school or high school, 2 indicating a lower academic degree, and 3 indicating an academic degree) to test the robustness of the results (Table 6). The potential confounding factors were added to the models in the first step, and the main predictors in the second step (Table 6). The predictive power (Nagelkerke R2) of the models is reported in Tables 5 and 6. For detailed estimates regarding the confounding factors, see Multimedia Appendix 1 (Tables S1-S16)."		
**8a) CONSORT: Method used to generate the random allocation sequence**		
Yes. This has been addressed in our manuscript's Methods section. "The participants (N=89) were randomized into 3 groups. The principal investigator of the study generated the random allocation sequence. Research nurses, who were in charge of scheduling laboratory visit times, randomly allocated the participants to groups after the participants agreed to voluntarily participate in the study. A blinded draw of a paper containing the numbers 1, 2a, or 2b was performed to randomly allocate participant to group 1 (the general guidelines group), 2a (the individualized intervention group), or 2b (the highly individualized intervention group)".		
**8b) CONSORT: Type of randomisation; details of any restriction (such as blocking and block size)**		
This has been described in the Methods section. See also our response to the CONSORT subitem 8a.		
**9) CONSORT: Mechanism used to implement the random allocation sequence (such as sequentially numbered containers), describing any steps taken to conceal the sequence until interventions were assigned**		
This has been described in the Methods section. See also our response to the CONSORT subitem 8a.		
**10) CONSORT: Who generated the random allocation sequence, who enrolled participants, and who assigned participants to interventions**		
This has been described in the Methods section. See also our response to the CONSORT subitem 8a.		
**11a) CONSORT: Blinding - If done, who was blinded after assignment to interventions (for example, participants, care providers, those assessing outcomes) and how**		
**11a-i) Specify who was blinded, and who wasn't**		
This has been described in the Methods section. See also our response to the CONSORT subitem 8a.		
**11a-ii) Discuss e.g., whether participants knew which intervention was the "intervention of interest" and which one was the "comparator"**		
**11b) CONSORT: If relevant, description of the similarity of interventions**		
Not applicable (NA) for the present study.		
**12a) CONSORT: Statistical methods used to compare groups for primary and secondary outcomes**		

Cardiorespiratory fitness (VO2max), the primary outcome of the MoMaMo! study will be reported elsewhere. The statistical methods have been reported in the manuscript's Methods section. Please find pages 6-7. "Statistical analyses focused on assessing attrition with respect to the participants' self-reported personality characteristics and psychological well-being at the 3-month follow-up. The analyses were conducted using SPSS versions 25 and 27 (IBM Corp). Descriptive statistics of the data are presented as frequencies, percentages, means, and standard deviations in Tables 1-4. The minimum and maximum values are also reported in Tables 2-4. The attrition analyses were conducted using hierarchical logistic regressions. Traditional statistical analysis methods have been used in previous longitudinal lifestyle interventions when examining attrition-related questions [36]. In the present study, the associations between the main predictors and the outcome, in other words, whether personality characteristics and indicators of psychological well-being contributed to attrition at the 3-month follow-up, were studied first (Table 5). Furthermore,the analyses were adjusted for the participants' sex, age, study group (group 1 vs group 2), and educational status (1 indicating vocational school or high school, 2 indicating a lower academic degree, and 3 indicating an academic degree) to test the robustness of the results (Table 6). The potential confounding factors were added to the models in the first step, and the main predictors in the second step (Table 6). The predictive power (Nagelkerke R2) of the models is reported in Tables 5 and 6. For detailed estimates regarding the confounding factors, see Multimedia Appendix 1 (Tables S1-S16)."		
**12a-i) Imputation techniques to deal with attrition / missing values**		
Yes, this paper focuses on participants' attrition from the program as has been stated in the manuscript's title. The statistical methods assessing the dropout have been presented in the manuscript's Methods section, pages 6-7. This study's results are presented in the pages 6-9. Please refer also to CONSORT subitem 12a above.		
**12b) CONSORT: Methods for additional analyses, such as subgroup analyses and adjusted analyses**		
Yes, the analyzing methods have been presented in the manuscript's Methods section, pages 6-7. "The attrition analyses were conducted using hierarchical logistic regressions." "Furthermore, the analyses were adjusted for the participant's sex, age, study group (group 1 vs group 2), and educational status (1 indicating vocational school or high school, 2 indicating a lower academic degree, and 3 indicating a higher academic degree) to test the robustness of the results (Table 6). The potential confounding factors were added to the models in the first step, and the main predictors in the second step (Table 6). The predictive power (Nagelkerke R 2) of the models is reported in Tables 5 and 6."		
RESULTS		
**13a) CONSORT: For each group, the numbers of participants who were randomly assigned, received intended treatment, and were analysed for the primary outcome**		
Yes, we have reported the number of participants in each group in the Methods and Results sections in the manuscript's pages 4-9. However, we analyzed the psychological factors contributing to participant attrition, not the primary outcome of the whole MoMaMo! intervention (cardiorespiratory fitness, VO2max) that will be reported elsewhere.		
**13b) CONSORT: For each group, losses and exclusions after randomisation, together with reasons**		
Yes, this has been reported in the flow diagram (Figure 1.).		
**13b-i) Attrition diagram**		
**14a) CONSORT: Dates defining the periods of recruitment and follow-up**		
Yes, these dates have been presented in the manuscript's Methods section. "Recruitment for the study started in April 2016 and the last follow-ups were performed in April 2020".		
**14a-i) Indicate if critical "secular events" fell into the study period**		
**14b) CONSORT: Why the trial ended or was stopped (early)**		
Not applicable (NA) for the present study.		
**15) CONSORT: A table showing baseline demographic and clinical characteristics for each group**		
Yes, please find Tables 1-4.		
**15-i) Report demographics associated with digital divide issues**		
Yes, the participants' demographics are presented in the Table 1.		
**16a) CONSORT: For each group, number of participants (denominator) included in each analysis and whether the analysis was by original assigned groups**		
**16-i) Report multiple "denominators" and provide definitions**		
Yes, please find the Tables 1-6, and the Results section in the manuscript.		
**16-ii) Primary analysis should be intent-to-treat**		
**17a) CONSORT: For each primary and secondary outcome, results for each group, and the estimated effect size and its precision (such as 95% confidence interval)**		
Yes, please find the Tables 5-6, and the Multimedia Appendix (Tables S1-S16). Please also see the manuscript's Results section.		
**17a-i) Presentation of process outcomes such as metrics of use and intensity of use**		
**17b) CONSORT: For binary outcomes, presentation of both absolute and relative effect sizes is recommended**		
Yes, please find the Tables 5-6, and the Multimedia Appendix (Tables S1-S16). Please also see the manuscript's Results section.		
**18) CONSORT: Results of any other analyses performed, including subgroup analyses and adjusted analyses, distinguishing pre-specified from exploratory**		
Yes, please find the Tables 5-6, and the Multimedia Appendix (Tables S1-S16). Please also see the manuscript's Results section.		
**18-i) Subgroup analysis of comparing only users**		
**19) CONSORT: All important harms or unintended effects in each group**		
No important harms or unintended effects were observed or reported.		
**19-i) Include privacy breaches, technical problems**		
**19-ii) Include qualitative feedback from participants or observations from staff/researchers**		
DISCUSSION		
**20) CONSORT: Trial limitations, addressing sources of potential bias, imprecision, multiplicity of analyses**		
**20-i) Typical limitations in ehealth trials**		
Yes, the study limitations have been considered in the manuscript's Discussion section. "This study focused on assessing the potential role of personality and psychological well-being in study attrition. Other factors, such as physical or social circumstances (eg, sudden outbreaks of disease or lack of social support) and those related to meeting goals were not assessed. Furthermore, specific study variables, including psychological well-being, were studied only in the beginning of the intervention. As psychological well-being, along with many other factors relating to human experience, fluctuates over time, it will be important to address the dynamic nature of this phenomenon in relation to study attrition (eg, using survival analyses) in future studies. Additionally, we cannot be absolutely certain whether the participants who did not come to the laboratory follow-up tests quit only the study or also their intervention program at the individual level. That is, they may or may not have continued changing or improving their lifestyles after they discontinued their participation in the MoMaMo! study. Although all the instruments' subscales were considered acceptable according to general rules, openness to experience had poor reliability (Cronbach α=.55). Some studies have demonstrated similar α values when optimal subset itemsreflecting personality dimensions were researched [54]. Generally, the α coefficient reliability has been high in this instrument's validation studies [21]. Furthermore, due to the relatively high attrition rate, some of the analyses performed in the study may lack statistical power. Consequently, these study results can be regarded only as directional. Studies with larger sample sizes and longer follow-up periods are needed to confirm our results."		
**21) CONSORT: Generalisability (external validity, applicability) of the trial findings**		
**21-i) Generalizability to other populations**		
**21-ii) Discuss if there were elements in the RCT that would be different in a routine application setting**		
**22) CONSORT: Interpretation consistent with results, balancing benefits and harms, and considering other relevant evidence**		
**22-i) Restate study questions and summarize the answers suggested by the data, starting with primary outcomes and process outcomes (use)**		
Yes, the results and interpretation have been considered in the manuscript's Discussion section." The health-technology assisted MoMaMo! study focused on physically inactive overweight and obese men and women who were at risk for a permanent physically inactive lifestyle and lifestyle-related chronic diseases. We focused on assessing whether participant personality characteristics and indicators of psychological well-being contributed to attrition within the MoMaMo! study and whether these factors should be taken into consideration more carefully in future interventions to support adults' commitment to technology-based intervention programs. Our results showed that 65 (73%) of the 89 initially recruited participants were still participating at the 3-month follow-up."		
**22-ii) Highlight unanswered new questions, suggest future research**		
Other information		
**23) CONSORT: Registration number and name of trial registry**		

Yes, the registration number and the name of the trial registry has been presented in the manuscript's method section, page 3 "MoMaMo! was registered at ClinicalTrials.gov (protocol record TYH2016215, NCT02686502)".		
**24) CONSORT: Where the full trial protocol can be accessed, if available**		
Yes, the trial can be found from the registry mentioned in the previous response. Please find the site Motivation Makes the Move! - Full Text View - ClinicalTrials.gov.		
**25) CONSORT: Sources of funding and other support (such as supply of drugs), role of funders**		
Yes, the information on the funding sources and other support is presented in the manuscript's Acknowledgement section. "The study was funded by Business Finland (575/31/2015), University of Helsinki, Ministry of Education and Culture (Finland),Helsinki Deaconess Institute, Diaconia University of Applied Sciences, Terveystalo Oyj (former Diacor Terveyspalvelut Oy), HUR Oy, Firstbeat Technologies Oy, Nukute Oy, Suunto Oy, Amer Sports Digital Services Oy, Nutri-Flow Oy, F8 Helsinki Oy, Diamond (TYH2016215), and Amer Cultural Foundation."		
**X26-i) Comment on ethics committee approval**		
**x26-ii) Outline informed consent procedures**		
**X26-iii) Safety and security procedures**		
**X27-i) State the relation of the study team towards the system being evaluated**		
